# Heterogeneous Multi-Layered Network Model for Omics Data Integration and Analysis

**DOI:** 10.3389/fgene.2019.01381

**Published:** 2020-01-28

**Authors:** Bohyun Lee, Shuo Zhang, Aleksandar Poleksic, Lei Xie

**Affiliations:** ^1^ Ph.D. Program in Computer Science, The City University of New York, New York, NY, United States; ^2^ Department of Computer Science, The University of Northern Iowa, Cedar Falls, IA, United States; ^3^ Ph.D. Program in Biochemistry and Biology, The City University of New York, New York, NY, United States; ^4^ Department of Computer Science, Hunter College, The City University of New York, New York, NY, United States; ^5^ Helen and Robert Appel Alzheimer’s Disease Research Institute, Feil Family Brain & Mind Research Institute, Weill Cornell Medicine, Cornell University, Ithaca, NY, United States

**Keywords:** data mining and knowledge discovery, machine learning, biological data analysis, biological network, link prediction, relation inference, deep learning

## Abstract

Advances in next-generation sequencing and high-throughput techniques have enabled the generation of vast amounts of diverse omics data. These big data provide an unprecedented opportunity in biology, but impose great challenges in data integration, data mining, and knowledge discovery due to the complexity, heterogeneity, dynamics, uncertainty, and high-dimensionality inherited in the omics data. Network has been widely used to represent relations between entities in biological system, such as protein-protein interaction, gene regulation, and brain connectivity (i.e. network construction) as well as to infer novel relations given a reconstructed network (aka link prediction). Particularly, heterogeneous multi-layered network (HMLN) has proven successful in integrating diverse biological data for the representation of the hierarchy of biological system. The HMLN provides unparalleled opportunities but imposes new computational challenges on establishing causal genotype-phenotype associations and understanding environmental impact on organisms. In this review, we focus on the recent advances in developing novel computational methods for the inference of novel biological relations from the HMLN. We first discuss the properties of biological HMLN. Then we survey four categories of state-of-the-art methods (matrix factorization, random walk, knowledge graph, and deep learning). Thirdly, we demonstrate their applications to omics data integration and analysis. Finally, we outline strategies for future directions in the development of new HMLN models.

## Introduction

A fundamental task in biological studies is to identify relations, more specifically dynamic functional associations or physical interactions between various chemical and biological entities. Network has been widely used to represent relations between entities in biology such as gene regulation, signaling transduction, metabolism, brain connectivity, and species interaction. In the network, a node represents an entity such as chemical compound, gene, protein, etc. A link between nodes represents their relations. There are basically two types of relations (or links), intra-domain relations and cross-domain relations. An intra-domain link denotes a relation between the same type of entities, e.g. a protein-protein interaction. A cross-domain link represents a relation between two entities that belong to different types, e.g. protein-chemical interactions. Given a network of nodes and links (observed relations), a computational challenge is how to predict missing relations.

**Figure 1:**
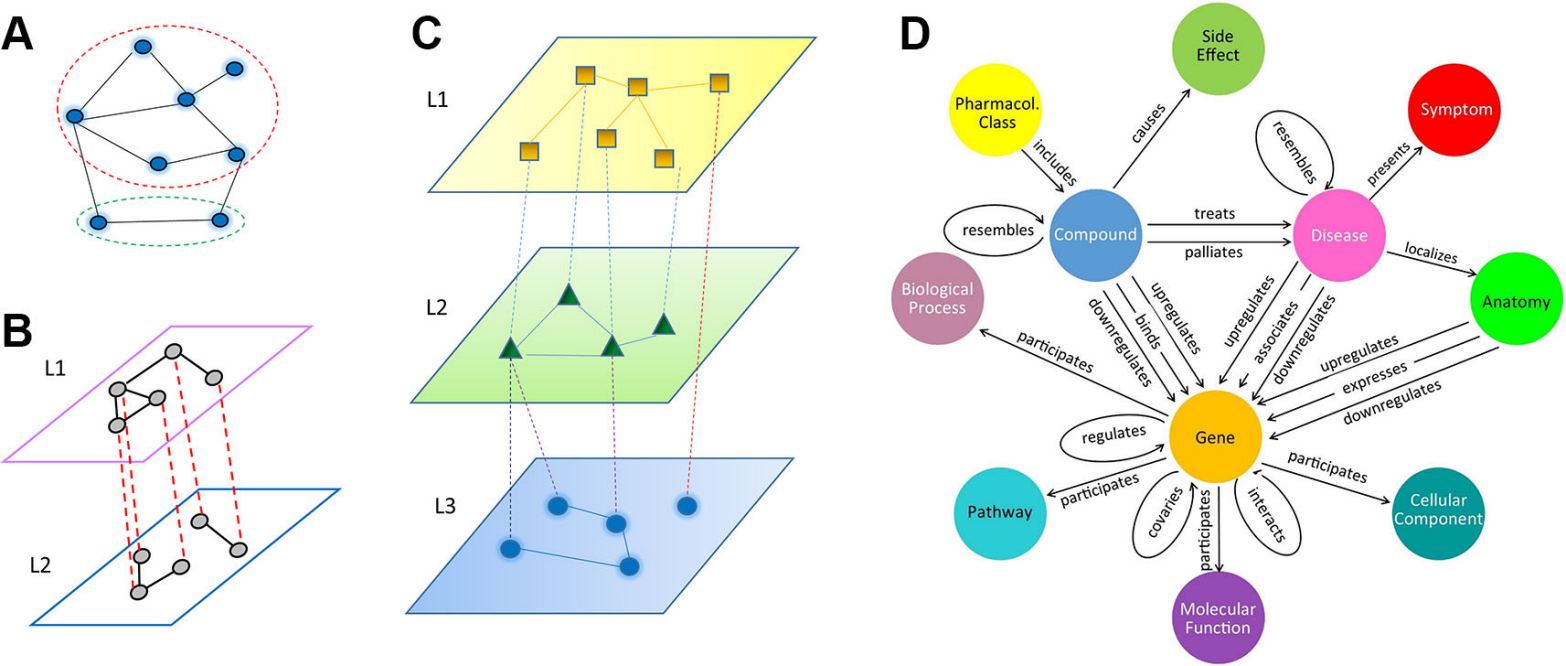
Illustration of three types of network models, **(A)** homogeneous network, where all nodes and edges are treated equally, even though they may belong to different types (dashed red and green circles). **(B)** multiplex network, **(C)** multi-layered network, **(D)** an example of heterogeneous multi-layered network HetioNet (Himmelstein and Baranzini, 2015).

Depending on the underlying algorithms, relation inference (or link prediction) can be formulated as a problem in a homogenous network, a multiplex network, or a heterogeneous multi-layered network (HMLN), as shown in **Figure 1**. In a homogenous network (**Figure 1A**), all nodes from different domains, as well as intra-domain and cross-domain relations, are treated equally. In contrast, multiplex and multi-layered networks separate different types of nodes and relations. A multiplex network is often used to represent homogeneous nodes that have different types of characterizations (a.k.a. views). For example, a gene can be characterized by multiple measurements of gene expression, essentiality, literature citation, phylogenetic profile, neighborhood in the interaction network, biological pathway involved, Gene Ontology annotation, protein domain profile etc. (Hwang et al., 2019). Each type of measurement can form a unique type of link between genes (**Figure 1B**). In a HMLN (**Figure 1C**), multiple types of heterogeneous nodes are involved. The nodes from each type are grouped into a single layer and treated separately. In the same vein, different types of intra-domain and cross-domain relations are marked differently in a multi-layered network. We note that more complex network representations, such as multiplex multi-layered network, may be needed in real applications. In this review, we focus on the cross-domain relation inference (or link prediction) problem for the HMLN. Readers can refer other excellent reviews of the multiplex networks (Chauvel et al., 2019).

Recently, multi-layered networks have been proposed to connect multiple inter-dependent heterogeneous domains in biology (Himmelstein and Baranzini, 2015; Chen et al., 2016; Kringelum et al., 2016; Li et al., 2017; Pinero et al., 2017) and ecology (Silk et al., 2018). A typical example of a multi-layered network is HetioNet (Himmelstein and Baranzini, 2015) (**Figure 1D**). HetioNet contains nine domains, namely compound, pharmacologic class, gene, pathway, biological process, disease, side effect, symptom, and anatomy. Another example of a multi-layered network is a multi-scale model that represents metabolic phenotypic response to vaccination (Li et al., 2017). It consists of four layers: blood transcriptomics, plasma metabolomics, plasma cytokines, and cell populations. The multi-layered network provides a natural way to represent the hierarchy of a biological system and its environmental context: from genetic markup to gene to biological pathway to cellular function to organismal phenotype to population dynamics. It allows us to uncover novel relations between biological entities (e.g. genotype-phenotype associations) on a multi-scale. Furthermore, the cross-layer relations may represent casual effects (e.g. loss-of-function mutation) rather than statistical correlations, e.g. Genome-Wide Association Studies (GWAS). Compared to a homogeneous single-layered network, a unique topological characteristic of a multi-layered network lies in its cross-layer relation or dependency structure in addition to intra-layer connectivity. For example, in HetioNet (Himmelstein and Baranzini, 2015), a compound can inhibit or activate a gene. This cross-layer dependency often plays a central role in a multi-layered network. The prediction of new cross-layer relations is often the key to new discoveries, such as a treatment of a new disease by an existing drug, i.e. drug repurposing.

Substantial efforts have been devoted to reconstructing a multi-layered network [e.g. HetioNet (Himmelstein and Baranzini, 2015)] from the experimentally observed or computationally inferred heterogeneous data sets. Even though the recent technology advances have enabled the generation of a vast amount of biological, physiological, and epidemiological data, the cross-layer relations observed by experiments are rarely complete, unbiased, and certain (Xie et al., 2017). Many important cross-layer relations are even completely missing. For example, there are no connections between genes and side effects in HetioNet, although such linkages are critical in understanding the molecular and genetic basis of adverse drug reactions. An unsolved computational problem is how to efficiently, accurately, and robustly infer the missing cross-layer relations in a HMLN.

In this review, we summarize the recent advances in the development of cross-layer relation inference algorithms for the HMLN, and their applications to biological discovery. The paper will be organized as follows. First, we will discuss the properties of biological HMLN. Second, we will introduce four major computational strategies for the cross-layer relation prediction, namely, matrix factorization, random walk, meta-path, and deep learning. Then, we will demonstrate the applications of these methods in biomedicine. Finally, we will discuss the unsolved issues and future directions.

## Characteristics of Biological HMLN

Biological HMLN has several unique characteristics that impose great challenges for cross-layer relation inference.

### Biasness

Due to limitation of experimental techniques and biases of researchers' interests, the observed data is highly skewed to certain gene families, species, diseases, etc. (Xie and Bourne, 2005) Rapid accumulation of large omics data could alleviate this problem to a certain degree. In addition, the reported positive results often greatly exceed the reported negative results, as the latter ones are seldom reported in the literature. Unless this reporting bias is taken into account, the models trained using the observed data by machine learning are unrealistic and hence unreliable when applied to unseen data.

### Noisiness

Many observed cross-layer links are noisy. The source of noisiness is mainly due to the inconsistency in the experimental and clinical observations. Given the same relation, the inconsistency might result from different experimental protocols, computational pipelines, and batch effects.

### Uncertainty

The relations in HMLN often come from calculated values or predictions made by heuristic algorithms. For example, many algorithms exist for computing intra-layer relations, such as chemical-chemical similarity. These methods differ in the choice of chemical representation and similarity metric employed. Similarly, no method is perfect for constructing cross-layer relations. While text mining is a popular technique, it is known to introduce a large number of false positives.

### Conditionality

Biological observations could be from different cell lines, culture conditions, disease conditions, and environmental conditions. Under different circumstances, the biological relations are changed dynamically. For example, the physical strength and functional consequence of protein-ligand binding are strongly dependent on that mutation and post-modification state of protein, gene expression profile, and other factors.

### Ambiguity

Many relations in HMLN are ambiguous and require proper classification. In one scenario, a relation can have opposite biological consequence. For example, the “association” relation between diseases and genes in HetioNet (**Figure 1D**) can be either “upregulate” or “downregulate”. Another example is the binding of bioactivity compounds on a protein. The bioactivity of compound is often ambiguous. It could be an agonist or an antagonist.

### Sparsity and Imbalance

The observed cross-layer links are highly sparse. In the real world, the number of relations of existence could be far less than the number of relations of non-existence. For example, a highly selective drug only binds to several protein isoforms among hundreds of thousands of protein isoforms in human. In addition, the observed relations are rare compared with the unobserved relations. For example, among hundreds of millions of sequenced genes, only tens of thousands of genes have the bioactivity data associated with chemical compounds. Because the negative cases often and greatly outnumber the positive ones, this imbalance imposes a great challenge in model training and evaluation.

### Open World Assumption

Missing links cannot be treated as false relations, but instead as “unknown”. In reality, these links could represent either a true or false relation (of different kinds, if the relation is not binary), or the lack of a relation.

## Algorithms for Relation Inference in HMLN

### Overview

**Figure 2:**
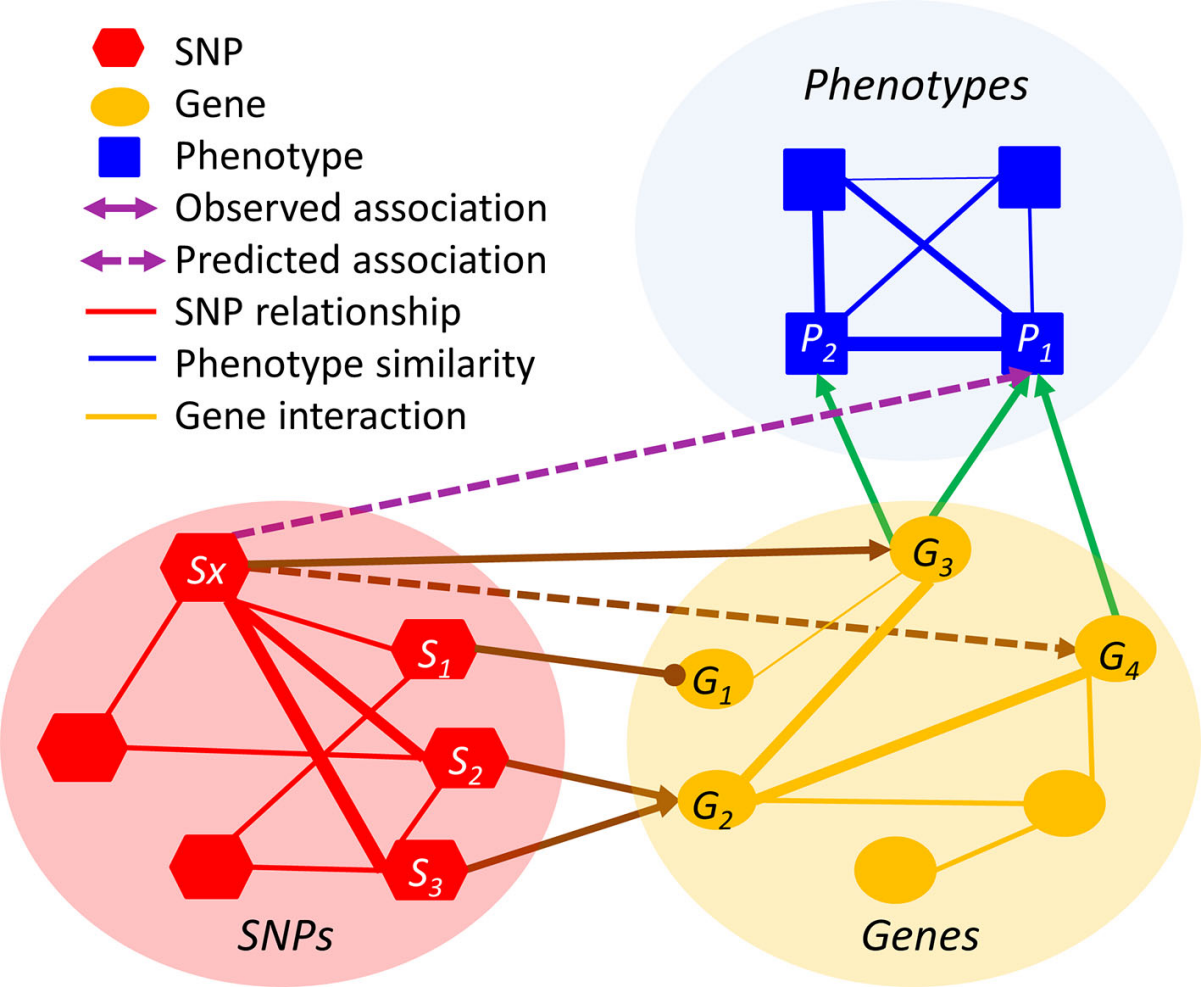
An illustration of relation inference in the HMLN. The line thickness is proportional to the degree of relation. Arrowed and headed lines denote positive and negative relations, respectively.

The premise of relation inference or link prediction is that the missing relations can be inferred from the existing observed relations. Although such direct linkages are sparse, they can be recovered through intermediate intra-domain and cross-domain relations. For example, if a rare SNP *S_x_* is a gain-of-function mutation of the gene *G_3_* and if *G_3_* is associated with the tall height *P_1_*, then *S_x_* is likely to be associated with *P_1_*, even if the *S_x_*-*P_1_* association is not statistically significant in the GWAS (**Figure 2**). However, such a simplistic inference method, based on the existing highly sparse and highly biased observations, is prone to type I errors. In the above setting, multiple genes (e.g. *G_3_* and *G_4_*) may be collectively responsible for *P_1_* and thus the likelihood of the inference “*S_x_* causes *P_1_*” has to be adjusted accordingly. To factor in the network multi-connectivity, an algorithm needs to jointly predict whether *S_x_* is associated with other genes and whether these genes are associated with *P_1_*, by simultaneously taking all observed cross-layer and intra-layer relations into account. In **Figure 2** example, the linkages of *S_x_- S_2_-*> *G_2_- G_4_* and *S_x_- S_3_-*> *G_2_- G_4_* will significantly strengthen the inferred *S_x_*-*P_1_* association.

**Figure 3:**
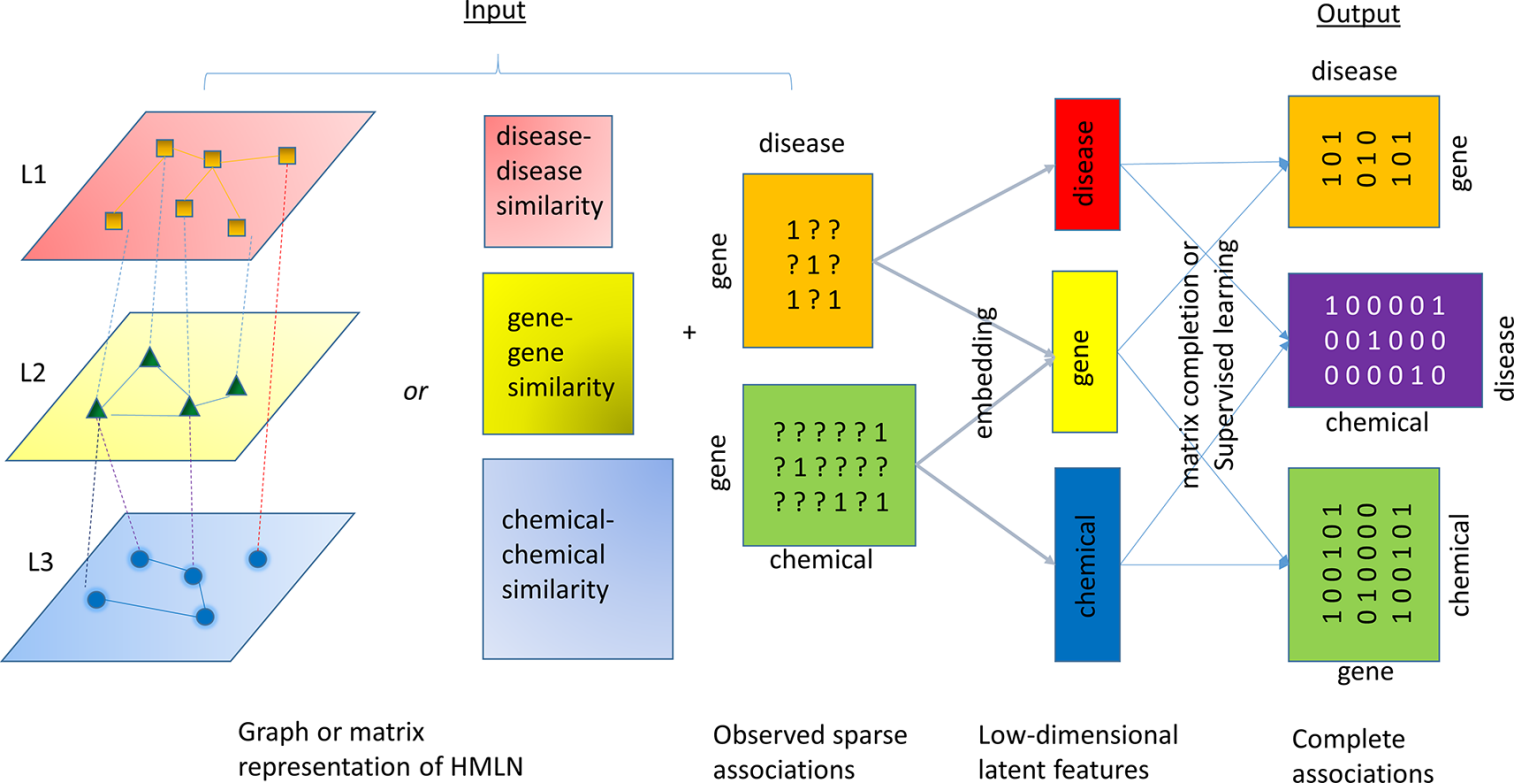
An illustration of the common algorithmic framework for the relation inference (link prediction). HMLN is represented as a graph or collection of matrices. An inference algorithm takes the HMLN as input and generates a low-rank latent feature representation of chemicals, genes, and diseases, respectively. The inner product of latent features or supervised learning techniques will reconstruct complete gene-disease, chemical-disease, and chemical-gene association matrix.

A number of algorithms have been developed to solve the relation inference problem in HMLN. All of these algorithms follow a common framework, consisting of two steps, as shown in **Figure 3**. The first step is to infer low dimension (i.e. rank) latent features for each entity and/or relation (aka node embedding and edge embedding). In the second step, the latent features from different layers are used to restore all missing cross-layer relations through a simple inner product or other more sophisticated machine learning techniques. In **Figure 3**, a chemical-gene-disease network is used to illustrate the concept. The input is a matrix representation of multi-layered network including both intra-layer relations (disease-disease similarity, gene-gene similarity, and chemical-chemical similarity) or their attributes (e.g. fingerprint representation for nodes in chemical layer, sequence representation for nodes in gene layer, and word2vec representation for nodes in disease layer), as well as a set of cross-layer relations (observed gene-disease association and chemical-gene interaction). In principle, even if we do not know any drug-disease associations, we can infer them through observed drug-gene, and gene-disease associations. The difference between the algorithms lies in the objective function for shallow or deep representations in the first step and machine learning methods for classification, regression, or ranking used in the second step.

In the next section, we review the major embedding algorithms in more details. These algorithms can be roughly classified into matrix factorization, random walk, meta-path, graph convolutional network (GCN), and their combinations.

### Matrix Factorization

The cross-layer relation inference problem is conceptually related to collaborative filtering (Goldberg et al., 1992). Commonly used collaborative filtering methods can be classified into two groups: neighborhood methods (Breese et al., 1998) and latent factor methods (Koren et al., 2009). As the latent factor approach is generally more effective in capturing the implicit cross-layer relations, many variants of this methodology, such as recommended systems (Portugal et al., 2018), have been proposed to address relation inference problems in a two-layered network (Gao et al., 2019; Xuan et al., 2019). However, few methods have been developed for the multi-layered network. Zitnik et al. developed a penalized matrix tri-factorization (PMTF) approach for data fusion (Zitnik and Zupan, 2015). Singh et al. proposed a collective matrix factorization (CMF) model to learn the dependencies across any two inter-dependent domains (Singh and Gordon, 2008). However, neither PMTF nor CMF takes the side information (i.e. intra-relations) into account. Moreover, both methods suffer the “cold-start” problem, which occurs when a new node arrives in the network.

Recently, Chen et al. developed the FASCINATE (Chen et al., 2016) algorithm to solve the multi-layered network inference problem, formulated as a weighted neighborhood-regularized collective one-class collaborative filtering problem. Mathematically, let **G** denotes a *g* × *g* layer-layer association matrix, where **G**(*i, j*) = 1 if layer-*j* associates with layer-*i*, and **G**(*i, j*) = 0 otherwise. Furthermore, let **A** = {A_1_, …,A_g_} represents a set of *g* within-layer connectivity matrices that describe the connectivity/similarity between nodes within the same layer. Finally, denote by **D** = {D*_i,j_ i, j* = 1,…, *g*} the set of cross-layer relation matrices, where D*_i,j_* specifies the relations between the nodes from layer *i* and the nodes from layer-*j*. (each relation is labeled 1, in case of an observed association; otherwise 0). The problem of inferring missing relations between layers is formulated as the following minimization problem:

(1) $$\underset{F_{i}\geq 0\left(i=1,\ldots ,g\right)}{\min}J=\underset{\text{Matching observed cross-layer relations}}{\underset{︸}{\sum_{i,j:G\left(i,j\right)=1}{\left\|W_{i,j}⊙\left(D_{i,j}-F_{i}{F^{\prime }}_{j}\right)\right\|}_{F}^{2}}}+\alpha \underset{\text{Node homophily}}{\underset{︸}{\sum_{i=1}^{g}tr\left({F^{\prime }}_{i}\left(T_{i}-A_{i}\right)F_{i}\right)}}+\beta \underset{\text{Regularization}}{\underset{︸}{\sum_{i=1}^{g}{\left\|F_{i}\right\|}_{F}^{2}}}$$

In the above loss function, *W_i,j_* denotes an *n_i_* × *n_j_* weight matrix that assigns different weights to different associations in the corresponding cross-layer relation matrix *D_i,j_*, depending on the confidence in *D_i,j_.* The confidence scores are extracted from the existing databases (Jensen et al., 2009; Kuhn et al., 2012). The matrix *F_i_* gives the low-rank representation for nodes in layer *i, while T_i_* is the diagonal degree matrix of A*_i_*. Overall, the first term in Eq. 1 is used to match all the cross-layer relations calibrated by the weight matrix *W_i,j,_*. The second term ensures that the similar nodes have similar low-rank representations. The third term is included to help prevent over-fitting. The optimization problem defined in Eq. (1) is non-convex. Block coordinate descent method is applied to find a local optima (where each *F_i_* naturally forms a ‘block’). Furthermore, the second term in Eq. (1) allows us to address the cold-start problem (namely the scenario where the query node does not have any known cross-layer links with the existing nodes in the network) based on similarity information.

There are several limitations of the existing MF-based methods for HMLN. First, the linear reconstruction of the complete matrix may not capture the complex cross-layer relations that are often non-linear. Deep neural network (DNN) has enjoyed great success in two-layered recommender system (Batmaz et al., 2019). Thus, it is interesting and tempting to extend the application of DNN to model the HMLN. Second, multiple types of links are often needed to model various biological relations between two layers. For example, there are three types of links between ‘gene’ and ‘disease’ in HetioNet: ‘down-regulate’, ‘up-regulate’, and ‘associate’. And, while ‘down-regulate’ and ‘up-regulate’ are mutually exclusive, ‘associate’ is ambiguous (could be either ‘down-regulate’ or ‘up-regulate’). Few of the existing MF-based methods can handle such multi-type relations. Finally, the scalability might become an issue when the existing implementations of MF are applied to extremely large matrices. A distributed variant of MF could alleviate the problem.

### Random Walk

Network propagation algorithm has been widely used in network biology (Cowen et al., 2017). Majority of applications of network propagation to biology networks are formulated in a homogeneous setting. For example, Lin et al. constructed a disease-gene-chemical network by integrating multiple data resources and then applied several homogenous network propagation algorithms for the relation inference (Lin et al., 2019). The random walk with restart (RWR) is one of the most representative network propagation algorithms. It was first developed to explore the global topology of networks, by simulating a particle that iteratively moves from a node to a randomly selected neighboring node (Lovasz, 1993). Only recently, random walk model has been extended to HMLN by allowing jumps across layers (Valdeolivas et al., 2019).

Consider an undirected graph, *G* = (*V*, *E*) with adjacency matrix *A*. An imaginary particle starts a random walk at the initial node *v*_0_ ∈ *V*. At a discrete time step *t* ∈ *N*, the particle is at node *v_t_*. Then, it walks from v*_t_* to *v_t+1_*, a randomly selected neighbor of v*_t_*, by following the transition matrix M calculated from A *via* column normalization (Lovasz, 1993). Probabilistically, ∀*x,y* ∈ *V,* ∀*t* ∈ *N*

(2) $$\text{P}\left(v_{t+1}=y|v_{t}=x\right)=\{\begin{array}{ll} & 1/d\left(x\right)\text{ if }\left(x,y\right)\in E\\ 0 & \text{otherwise}, \end{array}$$

where *d(x)* is the degree of *x* in the graph G. The probability distribution of random walk at time *t+1* is described by the following equation:

(3) $${\text{P}}_{t+1}^{\text{T}}={\text{MP}}_{t}^{\text{T}}$$

Accounting for the restart probability *r* on the seed node to avoid the particle’s dead-end, the random walk with restart (RWR) can be reformulated as:

(4) $${\text{P}}_{t+1}^{\text{T}}={\left(1-r\right)}^{*}{\text{MP}}_{t}^{\text{T}}+{\text{r}}^{*}{\text{P}}_{0}^{\text{T}}$$

Even a multiplex graph with the collection of *L* undirected graphs can be formulated as a RWR problem (De Domenico et al., 2013; Kivelä et al., 2014). Each layer α = 1,…,*L*, can be represented by an *n-by-n* adjacency matrix A^[α]^ = (A^[α]^ (*i,j*))*_i,j = 1,…,n_*, where A^[α]^ (*i,j*) = 1, if nodes i and j are connected in layer α, and 0 otherwise (Battiston et al., 2014). The multiplex graph is defined as G_M_ = (V_M_, E_M_), where:

(5) $${\text{V}}_{\text{M}}=\left\{v_{\text{i}}^{\alpha },i=1,\ldots ,n,\alpha =1,\ldots L\right\},\text{where }v_{\text{i}}^{\alpha }\text{stands for node }i\text{ in layer }\alpha ,\text{ and}$$

(6) $${\text{E}}_{\text{M}}=\left\{\left(v_{\text{i}}^{\alpha },v_{\text{j}}^{\alpha }\right),\;i,j=1,\ldots ,n,\;\alpha =1,\ldots L,\;{\text{A}}^{\left[\alpha \right]}\left(i,j\right)\neq 0\right\}\cup \left\{\left(v_{\text{i}}^{\alpha },v_{\text{i}}^{\beta }\right),i=1,\ldots ,n,\;\alpha \neq \beta \right\}.$$

The particle can walk from its current node $v_{\text{i}}^{\alpha }$ to any of its neighbors within a layer, or jump to any node $v_{\text{i}}^{\beta }$ with *α ≠ β* (De Domenico et al., 2013), and thereby travel from one layer to another, as shown in **Figure 1C**.

Extending classical RWR algorithm to a multiplex graph introduces a supra-adjacency matrix A of size nL*nL, which contains different types of transitions:

(7) $$A=\left(\begin{array}{cccc} \left(1-\delta \right)A^{\left[1\right]} & \frac{\delta }{\left(L-1\right)}\text{I} & \cdots & \frac{\delta }{\left(L-1\right)}\text{I}\\ \frac{\delta }{\left(L-1\right)}\text{I} & \left(1-\delta \right)A^{\left[2\right]} & \cdots & \frac{\delta }{\left(L-1\right)}\text{I}\\ \vdots & \vdots & \ddots & \vdots \\ \frac{\delta }{\left(L-1\right)}\text{I} & \frac{\delta }{\left(L-1\right)}\text{I} & \cdots & \left(1-\delta \right)A^{\left[L\right]} \end{array}\right)$$

In (7), I is the *n-by-n* identity matrix and A^[^ *^α^* ^]^ is the adjacency matrix of the layer *α*, as previously described. The diagonal elements represent potential intra-layer walks, whereas the off-diagonal elements account for possible jumps between different layers. The parameter δ ∈ [0,1] quantifies the probability of staying in the current layer or jumping to another layer. If δ = 0, the particle will stay in the same layer after a non-restart step.

Topological features of each node or edge derived from the RW algorithm can be directly applied to link prediction. Those features are often used as the basis of the more sophisticated node embedding algorithms, such as DeepWalk (Perozzi et al., 2014), Node2Vec (Grover and Leskovec, 2016), etc. However, these algorithms focus on the homogenous network and have not been extended to HMLN yet.

One of major limitations of the network propagation algorithm is that its performance strongly depends on the topology of the input network. It is less tolerant to biasness, noisiness, and incompleteness of the network, which are the characteristics of reconstructed biological HMLN.

### Meta-Path-Based Algorithms

Meta-path has been extensively studied in heterogeneous information networks (HIN) (Sun and Han, 2013). Since HMLN is a variant of HIN, the meta-path algorithm, described here, can be applied to the relation inference problem for HMLN. Given a directed graph representation: *G* = (*V*, *E*) of HIN, an object type mapping function τ: *V* → *A* and a link type mapping function φ : E → R are defined such that object *v* ∈ *V* belongs to one particular object type τ (v) ∈ *A* and each link e ∈ *E* belongs to a particular relation φ(e) ∈ *R*. A meta-path in *G* is a sequence of relations *R_1_*, …, *R_l_*, which connect two object types *A_i_* and *A_j_*. In the example of **Figure 2**, the relation types include SNP-associate-Phenotype (SaP), Chemical-associate-Gene (CaG), and Gene-associate-Phenotype (GaP), SNP-similar-SNP (SsS), Phenotype-similar-Phenotype (PsP), and Gene-similar-Gene (GsG). The SNP-Phenotype association between *S_x_* and *P_1_* can be defined by multiple meta-paths, e.g., SaG->GaP, SaG->GsG->GaP, and SsS->SaG->GaP, etc. By systematically designing meta-path based topological features and their measures in HLMN, supervised models can be used to learn the best weights associated with different topological features for effective relation inference (Sun et al., 2012). In general, for a target relation <*A_i_*, *A_j_*>, any meta-path starting with type *A_i_* and ending with type *A_j_* (other than the target relation itself) can be used as a topological feature. All these meta-paths can be obtained by traversing on the network schema, for example, using the breadth first search. Most algorithms for HIN reconstruction enumerate a predefined set of meta-paths. Once all meta-paths are defined, the next task is to design measures on their topology. The commonly used measures include the count of the path instances and the random walk-based measures. Using topological features, either a supervised or unsupervised learning model is used for node representation. For example, the metapath2vec method (Dong et al., 2017) uses a meta-path-based random walk to form the heterogeneous neighborhood of a node, taking advantage of word representation algorithm in the Nature Language Processing to perform node embedding (Dong et al., 2017). One of the drawbacks of these algorithms is that they require manual predefinition and enumeration of meta-paths. This may be not feasible for schema-rich HMLN or the relations that involve multiple hopping paths (Cao et al., 2017), e.g. relations inferred through thousands of similar chemicals.

### Graph Neural Network and Other Deep Learning Techniques

Besides the traditional algorithms, like matrix factorization, random walk, and meta-path, introduced in previous sections, the embedding of HMLN can also benefit from Deep Learning techniques, especially the Neural Networks (NNs). Though NNs are initially proposed to learn the embedding of data, such as texts, images, and videos, they have shown powerful performance when dealing with graph structured data, which exist in non-Euclidean domain. Due to the growing interests and demands in recent years, Graph Neural Networks (GNNs) have been proposed to learn the embedding of graphs (Li et al., 2015; Scarselli et al., 2008; Duvenaud et al., 2015; Kipf and Welling, 2017; Hamilton et al., 2017; Zhang et al., 2018; Ying et al., 2018; Morris et al., 2019; Xu et al., 2019; Zhang and Xie, 2019).

A GNN consists of a number of hidden layers that employ iterative, propagation procedures in order to transform different node and edge features. Each layer takes the output of the previous layer as the input. With graph structured data, GNNs adopt element (node or edge) features *X* and the graph structure *A* as input to learn the representation of each element *h*_*i*_, or graph *h*_*G*_, for different tasks. Each hidden layer employs the “aggregation” functions and the “update” functions (Battaglia et al., 2018). Each aggregation function *ρ* takes a set of node or edge features as input and reduces it to a single element which represents the aggregated information. The aggregations usually operate on the nearest neighbors or the local subgraphs of each element to capture local information gradually. Since the permutation invariance of the input holds in graph data, the *ρ* functions must also have the same property. These functions can take variable numbers of arguments. Commonly used *ρ* functions include sum (Xu et al., 2019), mean (Kipf and Welling, 2017), max-pooling (Hamilton et al., 2017) and attention mechanism (Velickovic et al., 2018; Wang et al., 2019; Fan et al., 2019). Update functions *ϕ* are applied across all elements to compute per-element updates after the aggregations. In the final layer, the generated embedding can be fed into the classification/prediction layer, and the whole model is trained for different (e.g. node classification, link prediction) tasks.

The design of GNNs is flexible. GNNs can be designed to fit different graph structures and different tasks. In the link prediction problem, the prediction of a feature (e.g. link or non-link) of a desired edge is based on the local structural information around that edge. For example, the method by Zhang et al. learns the link prediction heuristics from local (enclosing) subgraphs of edges rather than from the entire network (Zhang and Chen, 2018). The prediction of cross-layer relations follows a similar idea if HMLN is given as input. The model designed by Fan et al. learns the embedding of the two nodes by aggregating their neighbors (Fan et al., 2019). The embedding of two nodes is fed into a classification layer to classify the type of a given edge. Due to the topology of HMLN, GNNs can take meta-path into consideration when designing the aggregation functions *ρ*. In (Wang et al., 2019), the node embedding are computed by the neighbor nodes connected by meta-paths. During the training procedure, the effect of different meta-paths can be distinguished by using attention mechanism in aggregation. In (Zhang et al., 2018), the original input heterogeneous network is modified to be multi-channel network. Each channel is a homogeneous network consisting of the nodes that are connected by a similar type of meta-paths in the original network. Thus, GNNs can be used on each channel for learning the embedding, which is concatenated from all channels. As discussed in the previous section, the meta-path based GNN shares the same limitations of other meta-path based algorithms. New types of GNNs, those that explicitly take different types of relations into consideration, are needed for the link prediction problem in HMLN (Nathani et al., 2019).

Although Graph Neural Networks have been applied to heterogeneous networks and proven their ability of learning representations (Zhang et al., 2019), GNNs still exhibit limitations in several aspects. First, current GNNs proposed for the learning of heterogeneous networks do not particularly distinguish cross-layer from intra-layer relations. For example, while researchers can simply treat distinct relations as different types, the intra-layer relations in the same layer of an HMLN usually represent the similarity relation, which is semantically distinct from the cross-layer relation. The above needs to be taken into consideration when designing GNNs for HMLN. Second, the current design of GNNs relies on heuristics and empirical findings, which adds to the difficulty of learning the representations of HMLN. To enhance power of HMLN, it is crucial to properly identify the conditions that the aggregation and update functions ought to satisfy and to set those functions accordingly. Third, although GNNs can achieve promising results on different tasks for heterogeneous networks, it is hard for GNNs to have interpretability comparing to other traditional techniques. Therefore, new methods are needed to handle the problems that need interpretability (e.g. the need to find important nodes or edges that contribute to the results).

### Application of HMLN in Omics Data Integration and Analysis

Homogeneous and bi-layered network models have been widely applied in omics data integration and analysis. Recently, the HMLN has emerged as a powerful alternative. Here, we will highlight several exemplary applications of the HMLN to infer genotype-phenotype associations, and to predict chemical and other environmental perturbations.

Yao et al. integrated multi-omics data to construct a three-layered network model MetPriCNet, which consists of metabolite network, gene network, phenotype network, metabolite-phenotype network, metabolite-gene network, and gene-phenotype network (Yao et al., 2015). Afterwards, an RWR algorithm is applied to prioritize metabolites associated with diseases. The cross-validation on a benchmarking data set achieved the AUC values exceeding 0.9. An approach similar to MetPriCNet has been applied to identify and prioritize the metabolites responsible for atrial fibrillation (Yan et al., 2019), postmenopausal osteoporosis (Zhang et al., 2019), and Acute Lung Injury in Patients with Sepsis (Wang et al., 2019).

In addition to metabolite-disease association, the RWR method has been used to identify other molecular dysregulations that are associated with diseases based on the multi-layered network model. To infer disease associated m^6^A RNA methylation site, Tang et al. constructed a three-layered network, that includes a m^6^A site network, a gene network, a disease network, a m^6^A-gene network, and a gene-disease network (Tang et al., 2019). Xu and Wang applied random walk on a three-layer heterogeneous network that uses a kinase layer as an intermediate to infer disease-phosphorylation site relation. They showed that the three-layer phosphorylation site-kinase-disease network model is superior in inferring disease-phosphorylation site relation when compared with the existing random walk models and commonly used classification methods (Xu and Wang, 2016).

HMLN provides new opportunities for inferring novel drug-target-pathway-disease-side effect associations. The identification of such missing relations could facilitate the discovery of new therapies for complex diseases.

The ANTENNA method by Wang et al. employs a one-class collaborative filtering technique based on RWR and the matrix tri-factorization to predict the drug-disease associations using a three-layered drug-gene-disease network. In a comprehensive benchmarking study, ANTENNA outperformed the more conventional OCCF methods. Using ANTENNA, Wang et al. showed that diazoxide might inhibit the growth of triple negative breast cancer (TNBC) cells efficiently (Wang et al., 2018). Lim et al. applied FASCINATE to a three-layered drug-gene-side effect network model to identify biological pathways associated with rare side effects. Their predicted side effect-causing pathways are consistent with clinical evidences (Lim et al., 2018). Fu et al. extracted meta-path based topological features from a semantic network with nine object types (compound, ChEBI type, chemical substructure, protein, GO annotation, pathway, tissue, disease, and side effect), and twelve relation types. Subsequently, they applied the extracted features to predict drug-target interactions (Fu et al., 2016).

## Future Directions

### Representation of Biological Hierarchy and Environment

Only a few multi-layered network models for the genotype-phenotype associations have been developed that consist of more than three layers. These models lack the power to represent the full spectrum of information flow from the genotype to the phenotype. Even in a simplified picture, a multi-layered network model needs more than three layers to connect genome to phenome *via* epigenome (DNA layer), transcriptome (RNA layer), proteome (protein layer), and metabolome (metabolite layer). The representation of DNA, RNA, protein, and metabolite in the different layer could facilitate heterogeneous omics data integration and multi-scale modeling of information flow from genotype to phenotype. Furthermore, environmental components, such as gut microbiome, play a critical role in shaping the organismal phenotypes. With the exponential growth of different omics data from the same cohorts [e.g. TCGA (Cancer Genome Atlas Research Network et al., 2013)], the multi-layered network model represents a potentially powerful tool to integrate and analyze heterogeneous data sets for novel discovery.

### Incorporation of Mechanism-Based Modeling

The capability of data-driven modeling is limited by the existing data. We can enrich the missing relations in HMLN using complementary methods. For example, text mining is a commonly used tool to construct HMLN. Besides alternative machine learning approaches, mechanism-based modeling in biophysics, systems biology, and other fields can be applied to establish causal relations between entities. For example, protein-ligand docking can be applied to infer chemical-protein interactions. The mechanism argument HMLN may provide us with new opportunities for novel discovery, as demonstrated in a recent study (Lim et al., 2019). However, the potential false positives from the outside predictions should be taken into consideration when designing HMLN learning procedures.

### Data Consolidation and Normalization

When reconstructing HMLN, both intra-layer and cross-layer relations can come from multiple resources. For instance, in HumanNet, gene-gene co-functional links are derived from co-citation, co-essentiality, co-expression, pathway database, protein-domain profile association, and gene neighborhood (Hwang et al., 2019). Another example is chemical-protein interaction (Gaulton et al., 2012). The binding assay could be performed using different experimental techniques, and measured by different metrics (IC50, pKi, etc.). However, mapping the entities, minimizing batch effects, and normalizing the weights of different edge types in the same network remain the challenging tasks.

### Inference of Directionality and Trend of Relations

Few of relation inference algorithms can predict the directionality and trend of edges. The directionality means that one entity has effect on another but not vice versa. The trend represents distinct and often opposite functional consequence. For example, a drug can down- or up-regulate a gene. The identification of the directionality and trend of relation is pivotal to understand many biological processes such as drug action, signaling transduction and gene regulation, and determine causality between biological entities. For example, knowing that a chemical *C* interacts with a gene *G*, which is associated with a disease *D*, does not necessarily imply that the compound *C* will be effective on the disease *D*. On the other hand, if the compound *C* up-regulates the gene *G*, and the gene *G* is down-regulated in the disease *D*, than it is more likely for the compound *C* to treat or palliate the disease *D*. Recent development on signed network algorithm may provide partial solution to this problem (Kim et al., 2018).

### Inference of Non-Binary and Dynamic Relations

Existing link prediction algorithms for HMLN mainly focus on binary relations. However, other types of relations, such as unary and higher-arity relations, are needed to encode more complete biological knowledge. The unary relation represents the property of an entity, for example, the expression value of a gene. When modeling a dynamic system, a relation is associated with time and location. A single binary relation is not sufficient to capture its temporal and spatial nature. In this case, the higher-arity relations might prove beneficial. An example of a ternary relation is “gene *A* with a mutation *M* down-regulates the expression of gene *B* in neuro cells”. This relation includes three entities or layers (mutation, gene, and cell), and it can be expressed by three binary relations: “Mutation *M* is in gene A”, “drug *A* down-regulates gene *B*”, and “gene *B* is expressed in the neuro cells”. However, the genes *A* and *B* might be expressed in other types of cells in addition to the neuro cells. The mutation *M* in gene *A* may not down-regulate gene *B* in other cells. As a result, the tissue-specific correspondence between mutation *M* and the neuro cell is lost.

### Incorporation of Ontology

A number of biomedical ontologies have been developed to facilitate knowledge integration and discovery (Musen et al., 2012). These ontologies can serve as HMLN constraints to reduce false positives and resolve contradictory relations. There are two types of ontology constraints that can be applied to HMLN, namely deterministic constraint and functional constraint. A deterministic constraint imposes a clear dependency on relations such as “IsA” and “LocatedIn”. For example, if a protein binds to zinc, it is safe to state that the protein is metal-binding, because zinc is a metal. One can precompute all relations derived from the deterministic constraint and add them to HMLN prior to learning. Functional constraints enforce mutual exclusiveness between possible values. For example, if a chemical *A* is a known inhibitor of enzyme *B*, one can exclude the relation “*A* activates *B*” from HMLN.

### Sampling of Negative Relations

Many learning algorithms need a balanced number of negative examples. As mentioned in section 2, there are much less negative examples than positive examples in the biological HMLN, although, in reality, the negative cases are substantially more frequent than the positive ones. The conventional method is to randomly sample from a uniform distribution after excluding positive examples. However, this approach may not be applicable to the biological HMLN, where opposite relations exist between two entities. For example, a drug can either “down-regulate” or “up-regulate” a gene. It is not obvious how to assign the sampled relations to the opposites of “down-regulate” or “up-regulate”. Cai et al. have recently developed an adversarial reinforcement learning framework to assign the negative samples (Cai and Wang, 2017). This approach can be extended to the negative sampling for different relation types in HMLN.

### Visualizing HMLN

Visualization plays a key role in data mining tasks. Although many computational platforms, such as Cytoscape (Shannon et al., 2003), have been developed for the network visualization, few tools are available for efficient and intuitive visualization of HMLN, especially when the network is large (Mcgee et al., 2019). There is an urgent need to design a robust data structure for the representation and grouping of nodes and relations in HMLN in a way that they can be efficiently mapped to the graphic user interface and easily navigated by users.

## Author Contributions

BL, SZ, AP, and LX wrote the manuscript.

## Funding

This work was supported by Grant Number R01LM011986 from the National Library of Medicine (NLM), Grant Number R01GM122845 from the National Institute of General Medical Sciences (NIGMS), and Grand Number R01AD057555 of National Institute on Aging on the National Institute of Health (NIH). The funding agencies do not play roles in writing this manuscript.

## Conflict of Interest

The authors declare that the research was conducted in the absence of any commercial or financial relationships that could be construed as a potential conflict of interest.
